# Immune Reconstitution Syndrome Caused by Nontuberculous Mycobacteria: A Case Report and Review of Literature

**DOI:** 10.7759/cureus.64146

**Published:** 2024-07-09

**Authors:** Akshay Kohli, Shadee Tajik, Omar Abdulfattah

**Affiliations:** 1 Pulmonary and Critical Care Medicine, Southern Illinois University School of Medicine, Springfield, USA; 2 Internal Medicine, Southern Illinois University School of Medicine, Springfield, USA; 3 Critical Care, Southern Illinois University School of Medicine, Springfield, USA

**Keywords:** infectious disease medicine, immune repose, hiv diseases, nontuberculous mycobacteria (ntm), immune reconstitution syndrome

## Abstract

Immune reconstitution inflammatory syndrome (IRIS) is a potentially life-threatening phenomenon associated with the initiation of antiretroviral therapy in patients with acquired immunodeficiency syndrome due to a human immunodeficiency virus (HIV) infection. It is thought to be an exaggerated inflammatory response to an existing pathogen or even its antigen. We present a case of IRIS due to a non-tuberculous mycobacteria infection in a young patient with HIV infection who was recently started on therapy. This case highlights the challenges of making such a diagnosis and the importance of multidisciplinary team discussions with pulmonary and infectious diseases for optimal management of these patients.

## Introduction

Immune reconstitution inflammatory syndrome (IRIS) is a rare but potentially life-threatening phenomenon that typically occurs in HIV-infected individuals shortly after initiating highly active antiretroviral therapy (HAART). The etiology behind this condition is the recovery of the body's immune system and becoming capable of mounting an inflammatory response against previously acquired infections or antigens.

It is estimated that about 10-25% of patients who start HAART experience IRIS [1-3]. HAART therapy increases CD4+ T lymphocytes and thus boosts the adaptive immune system. This leads to a paradoxical worsening of the clinical condition due to an exaggerated inflammatory response or the unmasking of a previously subclinical infection. Risk factors for the development of IRIS include a rapid decline in viral load (especially in the first three months), a low baseline CD4 count, and HAART-naive patients [2,4]. An inflammatory reaction may be to a viable or non-viable pathogen or even to its residual antigen. Common infections include mycobacterium, herpesvirus, cytomegalovirus, and varicella zoster [2,3,5]. Among the mycobacteria, *Mycobacterium tuberculosis* is the most common culprit; however, non-tuberculous mycobacteria (NTM) have also been reported [6].

*Mycobacterium avium complex* (MAC) is a gram-positive, non-spore-forming acid-fast bacillus found ubiquitously in nature and is acquired via inhalation or ingestion. The bacteria infect macrophages and are carried via the lymphatic system to other parts of the body. It can cause various clinical manifestations, such as pulmonary disease, skin and soft tissue disease, musculoskeletal diseases, and even disseminated disease. In immunocompromised patients, disseminated MAC infections usually occur when CD4 counts are <50 cells/microliter. In patients without a history of HIV, risk factors for disseminated MAC infection include chronic lung disease, such as bronchiectasis [7,8].

We present an interesting case of IRIS presenting as pulmonary NTM in a young patient with HIV infection who recently started on HAART.

## Case presentation

**Figure 1:**
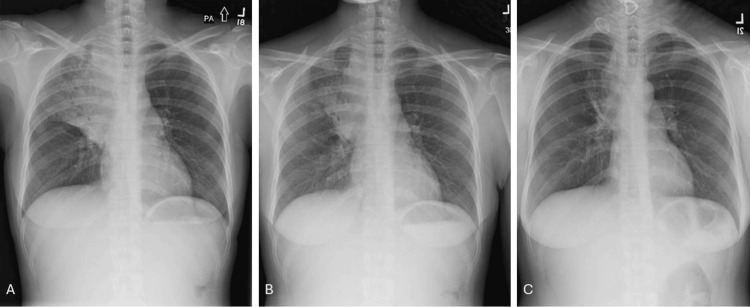
A: Chest radiograph (PA view) on admission shows right upper lobe consolidation. B: Chest radiograph (PA view) one month after starting MAC therapy. C: Chest radiograph after completion of therapy.

A 29-year-old woman with a past medical history of HIV presented to the emergency department (ED) with complaints of hemoptysis, cough, night sweats, and perianal itching for two weeks. She had been started on HAART four months before her presentation with Tivicay (Dolutegravir) 50 mg and Descovy (Emtricitabine and Tenofovir Alafenamide) 250-20 mg daily. Of note, she had no prior positive cultures for NTM. Her vitals on admission revealed a blood pressure of 94/60mmHg with a heart rate of 114/minute. She was afebrile, and her oxygen saturation was 98% on room air. Labs were significant for normocytic anemia with a hemoglobin of 11.3 gm/dL and an elevated neutrophil-to-lymphocyte ratio. Her CD4 count was 337 (the CD4 count at the time of HAART initiation was 54). The rest of the labs were unremarkable. A chest X-ray demonstrated new lobar pneumonia in the right upper lobe (RUL) (Figure 1A). 

**Figure 2:**
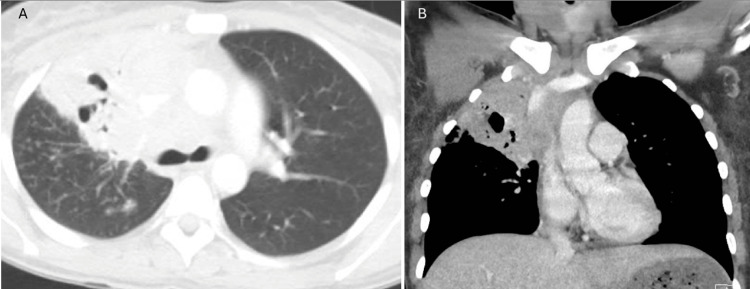
CT scan lung window axial view (Panel A) showing right upper lobe involvement with air bronchograms, and mediastinal window in coronal view (Panel B) showing soft tissue involvement within the mediastinum and right hilar region.

Chest CT was significant for a new large consolidation within the right upper lobe with air bronchograms, as well as hyper-density and soft tissue involvement within the mediastinum and right hilum (Figure 2).

Infectious workup with *streptococcal* and *legionella* urine antigens, beta-d-glucan, fungal serology, quantiferon, HIV viral load, CD4 count, sputum with AFB stain x3, as well as *Pneumocystis jirovecii* pneumonia (PJP) stain on sputum and blood cultures were all negative. HAART and PJP prophylactic medications were resumed. Broad-spectrum antibiotics, including vancomycin, cefepime, and azithromycin, were started. Bronchoscopy with bronchoalveolar lavage (BAL) of RUL was performed and sent for cell count, gram stain, culture, viral smear, AFB analysis, and cytology.

The patient’s clinical condition improved over the course of her hospitalization, and she was discharged with a follow-up scheduled for infectious disease and pulmonology. The BAL cultures were positive for *Mycobacterium avium* complex, and an endobronchial biopsy revealed granulomatous inflammation. A diagnosis of MAC infection was made, and she was treated with Azithromycin 500 mg, Ethambutol 800 mg, and Rifampin 600 mg, to be continued for one year following negative sputum mycobacterial cultures. HAART therapy was transitioned to Truvada (Emtricitabine and Tenofovir Disoproxil Fumarate) from Descovy (Emtricitabine and Tenofovir Alafenamide) due to drug interactions, and her Tivicay (Dolutegravir) was increased to 50 mg BID. Her symptoms significantly improved, and after three months of treatment, her sputum cultures were negative for AFB. Her post-treatment radiograph revealed a decrease in the infiltrates (Figures 1B, 1C). 

## Discussion

Multiple organisms can present as manifestations of IRIS, and because of that, it can be difficult to standardize a definition. In general, the infectious etiologies commonly reported in IRIS are cytomegalovirus (CMV), herpes virus (HSV), *cryptococcus*, and *Mycobacterium tuberculous* complex. Research into the pathophysiological mechanisms of IRIS is limited; however, there is some data to suggest that certain cytokines play a role. One study found that certain proinflammatory cytokines, such as IL-6 levels and CRP, are transiently elevated during an IRIS event [9]. In this study, blockage of IL-6 in mice induced with IRIS led to prolonged survivability and prevented wasting disease. It is generally accepted that the low amount of pathogen-specific CD4 T cells leads to the overgrowth of pathogens and triggers an overactive innate immune response [10]. This suggests that the adaptive as well as innate immune responses may play a role in the development of IRIS.

There is no specific test for a definitive diagnosis of IRIS, making the diagnosis challenging and thus requiring a high index of suspicion. It is important to differentiate between IRIS and ART failure [11]. The current diagnostic criteria for IRIS require the following: Presence of an atypical opportunistic infection, a decrease in the level of HIV RNA by at least 1 log_10_ copies/ml, an increased blood CD4+ T-cell count after HAART, and an increase in immune response to a specific pathogen. However, the diagnosis of IRIS remains challenging due to the complexity of the syndrome and the lack of a definitive test to confirm the diagnosis [11,12]. Differentiating IRIS from ART failure is not easy and is mainly based on clinical judgment. Moreover, in ART failure, there is an increase in viral load and a low CD4 count. Table 1 outlines the difference between IRIS and ART failure. 

**Table 1:** Differentiating immune reconstitution inflammatory syndrome from antiretroviral therapy failure HIV: Human immunodeficiency virus, NSAIDS: Non-steroidal anti-inflammatory drugs

	Immune reconstitution inflammatory syndrome (IRIS)	Antiretroviral therapy failure (ART failure)
Definition	Immune mediated hyperinflammatory response secondary to recovering immune system in the immunocompromised (HIV, rheumatoid arthritis, cancer patients).	Treatment failure can be defined as progression of the disease after initiation of Highly active Antiretroviral Therapy (HAART).
Pathogenesis	Immune response to previously acquired infections, antigens, or new infections.	Causes include poor adherence, drug resistance, poor absorption of medication, inadequate dosing and drug-drug interactions.
Onset	Typically, a few weeks after starting antiretroviral therapy.	Variable, usually within a few months of starting therapy.
Symptoms	Fever, lymphadenopathy, Exacerbations of previous infections.	New HIV-related opportunistic infections that are not related to IRIS, pre-existing organ damage or another cause.
Diagnosis	Clinically diagnosed. In HIV positive patients, the patient should be receiving ART therapy with either a decrease in HIV-1 RNA level from baseline or an increase in CD4+ cells from baseline or both.	Virologic failure is defined as viral load >200 copies/mL after 6 months of therapy. Drug-resistance testing should be performed while the patient is taking the failing regimen.
Treatment	Treat underlying infections, anti-inflammatory medications (steroids, NSAIDs); Continue ART therapy.	New regimen should include at least two fully active drugs.

The diagnostic workup for MAC, as recommended by the IDSA, includes clinical, radiological, and microbiological evidence in order to diagnose an active infection versus environmental contaminants. Current guidelines recommend greater than one positive sputum culture, with the same species being isolated in greater than or equal to two sputum cultures. Other acceptable evidence includes a positive bronchial wash, a BAL culture, or a transbronchial vs. lung biopsy. There will also need to be clinical signs and symptoms with radiological evidence of nodular or cavitary opacities on CXR or bronchiectasis with numerous nodules on CT [13].

Although not typically rare, MAC-related IRIS in a patient who has no history of MAC-opportunistic infection is difficult to diagnose. In a study looking at the incidence of IRIS when HAART is initiated after an AIDS-defining opportunistic infection, 11% (21 out of 196 patients) developed paradoxical IRIS in the first year of HAART. In the same study, only 1 out of 10 patients suspected to have MAC-associated IRIS met the predefined criteria of IRIS [14]. MAC-associated IRIS usually develops in patients with an initial CD4 count <100 cells/µl and manifests after 2-8 weeks of ART. Common manifestations include lymphadenitis or pulmonary disease; contrary to opportunistic MAC infection, disseminated infection is less common in MAC-associated IRIS [6,15,16].

In our review of the literature (Table 2), the patients commonly presented with symptoms such as fever, dyspnea, cough, and hemoptysis. There were reports of mediastinal and hilar lymphadenopathy as well. Potential risk factors associated with the development of MAC-associated IRIS are elevated ALP levels and increased CD8 + T-cell activation with low CD4 counts at ART initiation [17]. Interestingly, our patient had normal ALP levels on ART initiation, but her CD4 count was low at 58 cells/μl.

**Table 2:** Previous cases with similar presentations had MAC-associated IRIS after the initiation of ART. MAC: Mycobacterium avium complex; IRIS: Immune reconstitution inflammatory syndrome, ART: Antiretrovial therapy, Anti-MAC therapy: a macrolide plus ethambutol plus a rifamycin

Author	No. of patients	Presenting symptoms	Organism	Treatment	ART initiation to onset of IRIS
French et al., 2000 [3]	5	Fever and Lymphadenopahty	M. avium	Anti-MAC therapy	Unknown
Namkoong et al., BMC Med Imaging, 2015 [23]	1	Mediastinal and Hilar lymphadenopathy, bilateral pulmonary infiltrates.	MAC	Rifampicin, ethambutol, clarithromycin, and levofloxacin	5 months
Sohn S et al. 2018 [24]	1	Dyspnea	MAC	Rifampicin, ethambutol, clarithromycin, levofloxacin	16 days
Harshkumar P et al., 2022 [25]	1	Fevers and Tachycardia B/l mediastinal and hilar lymphadenopathy	MAC	Anti-MAC therapy	6 weeks
Nabeya et al., 2022 [26]	1	Hemoptysis and dyspnea, right upper lobe consolidation	MAC	Anti-MAC therapy	9 months
Xerri et al., 2021 [27]	1	Chest pain, dry cough, fever, lymphadenopathy	MAC	Anti-MAC therapy	3 weeks
Curic et al., 2016 [28]	1	Cough, Malaise, Progressive Dyspnea, Fevers, Bilateral Infiltrates	MAC	Anti-MAC therapy	3 months

Previously, prophylaxis for MAC with weekly azithromycin was recommended in patients with CD4 cell counts less than 50/µL. However, newer evidence suggests the risk for MAC is low among these patients taking ART. Therefore, primary MAC prophylaxis is no longer recommended in newly diagnosed patients prescribed ART [18]. Treatment guidelines for pulmonary MAC infection in patients with cavitary lesions or severe bronchiectatic nodular disease include a daily regimen of azithromycin, rifampin, and ethambutol. International guidelines recommend treatment until sputum cultures have been continuously negative for at least 12 months. Aminoglycosides such as amikacin can be used in cases of macrolide-resistant MAC. Surgical treatment is reserved for patients with localized fibro-cavitary disease, especially those with macrolide-resistant disease or who fail medical therapy [13].

Treatment of IRIS depends on the severity of symptoms and the inflammatory response. Non-steroidal anti-inflammatory drugs can be used in mild cases to reduce the symptoms of an inflammatory reaction. Corticosteroids are recommended for severe or life-threatening reactions, except in IRIS caused by Kaposi's sarcoma or *Cryptococcus*, where steroids are better avoided. Surgical resection in the form of lobectomy or segmentectomy has been used as an adjunct to medical therapy in patients with focal disease. Supportive measures depend on the system involved in the reaction. ART generally should be continued; however, it is recommended to hold ART therapy in life-threatening cases, such as those with a risk of central nervous system involvement, which includes progressive multifocal leukoencephalopathy [19-22].

## Conclusions

IRIS presenting in an HIV/AIDS patient as a NTM infection is infrequently seen. It is important for physicians to identify latent infections before initiating HAART therapy and to be aware of the signs and/or symptoms of NTM infections in at-risk patients.
